# Application of Silica Nanoparticles in Combination with Two Bacterial Strains Improves the Growth, Antioxidant Capacity and Production of Barley Irrigated with Saline Water in Salt-Affected Soil

**DOI:** 10.3390/plants11152026

**Published:** 2022-08-03

**Authors:** Khadiga Alharbi, Emadeldeen Rashwan, Hossam Hussein Mohamed, Abdelmoniem Awadalla, Alaa El-Dein Omara, Emad M. Hafez, Tarek Alshaal

**Affiliations:** 1 Department of Biology, College of Science, Princess Nourah Bint Abdulrahman University, Riyadh 84428, Saudi Arabia; 2 Agronomy Department, Faculty of Agriculture, Tanta University, Tanta 31527, Egypt; emad.rashwan@agr.tanta.edu.eg; 3 Department of Agronomy, Faculty of Agriculture, Ain Shams University, Cairo 11782, Egypt; dr.hossam16@yahoo.com; 4 Department of Agronomy, Faculty of Agriculture and Natural Resources, Aswan University, Aswan 81528, Egypt; abdelmoniemomr@yahoo.com; 5 Department of Microbiology, Soils, Water and Environment Research Institute, Agricultural Research Center, Giza 12112, Egypt; alaa.omara@yahoo.com; 6 Department of Agronomy, Faculty of Agriculture, Kafrelsheikh University, Kafr El-Sheikh 33516, Egypt; 7 Department of Applied Plant Biology, Institute of Crop Sciences, University of Debrecen, Böszörményi Street 138, 4032 Debrecen, Hungary; 8 Soil and Water Department, Faculty of Agriculture, Kafrelsheikh University, Kafr El-Sheikh 33516, Egypt

**Keywords:** cereal crops, nanoparticles, PGPR, plant defense system, yield, salinity, irrigation

## Abstract

Exploitation of low-quality water or irrigation of field crops with saline water in salt-affected soil is a critical worldwide challenge that rigorously influences agricultural productivity and sustainability, especially in arid and semiarid zones with limited freshwater resources. Therefore, we investigated a synergistic amendment strategy for salt-affected soil using a singular and combined application of plant growth-promoting rhizobacteria (PGPR at 950 g ha^−1^; *Azotobacter chroococcum* SARS 10 and *Pseudomonas koreensis* MG209738) and silica nanoparticles (SiNPs) at 500 mg L^−1^ to mitigate the detrimental impacts of irrigation with saline water on the growth, physiology, and productivity of barley (*Hordum vulgare* L.), along with soil attributes and nutrient uptake during 2019/2020 and 2020/2021. Our field trials showed that the combined application of PGPR and SiNPs significantly improved the soil physicochemical properties, mainly by reducing the soil exchangeable sodium percentage. Additionally, it considerably enhanced the microbiological counts (i.e., bacteria, azotobacter, and bacillus) and soil enzyme activity (i.e., urease and dehydrogenase) in both growing seasons compared with the control. The combined application of PGPR and SiNPs alleviated the detrimental impacts of saline water on barley plants grown in salt-affected soil compared to the single application of PGPR or SiNPs. The marked improvement was due to the combined application of PGPR and SiNPs, which enhanced the physiological properties (e.g., relative chlorophyll content (SPAD), relative water content (RWC), stomatal conductance, and K/Na ratio), enzyme activity (superoxide dismutase (SOD), catalase (CAT), and peroxidase (POX)), and yield and yield-related traits and nutrient uptake (N, P, and K) of barley plants. Moreover, the Na+ content, hydrogen peroxide (H_2_O_2_) content, lipid peroxidation (MDA), electrolyte leakage (EL), and proline content were reduced upon the application of PGPR + SiNPs. These results could be important information for cultivating barley and other cereal crops in salt-affected soil under irrigation with saline water.

## 1. Introduction

Barley (*Hordeum vulgare* L.) is an established cereal crop in the world. The global production volume of barley amounted to about 147.05 million metric tons in the 2021/2022 crop year, decreasing from around 160.53 million metric tons in 2020/2021 [1]. It has been a well-known food component since the era of Sumerians [2]. Recently, it has been used as the main ingredient in animal feed, malting, brewing, and biodiesel production [3]. However, its nutritional value is high, and barley could also be a crucial food crop [4]. Barley can grow well in the North Coastal Region in Egypt and newly reclaimed soils, especially when grown under water-stressed conditions due to its remarkable tolerance of abiotic stress [5]. However, knowledge about its response to low-quality irrigation water in salt-affected soil is limited.

World agriculture is constantly subjected to many environmental challenges related to climatic changes, such as water shortage, saline and salt-affected soil, and poor-quality irrigation water [6,7]. More than 800 million ha of the world’s lands are saline soil, either by salinity (397 Mha) or sodicity (434 Mha) [8]. Salt stress negatively impacts water and ion movement from the soil solution through the root hairs to plant leaves, resulting in a reduced growth rate, shorter height, and sometimes fewer leaves [9]. Salinity effects on yield pose an additional risk in arid and semi-arid regions due to a lack of rainfall, higher temperature, poor water quality, and poor soil management practices [10]. Hafez et al. [11] stated that the increase in the salinity level of irrigation water led to a significant decline in the uptake of N, P, and K in barley. Likely, the contents of Ca, Mg, and N in plant tissues declined after increasing the NaCl content in the irrigation water [12]. Additionally, salinity injures the plant by decreasing the leaf water contents, nutrient balance, development, and plant productivity [13]. Salinity stress restrains plant growth due to its negative impacts on biochemical and physiological attributes [14] due to high osmotic pressure and ion toxicity (elevated Na^+^ and Cl^−^ ions), which is considered a limiting factor of the sustainability of agricultural production [15]. Consequently, the limited resources of fresh water, particularly in arid and semi-arid regions, drive farmers towards the engagement of low-quality water for the irrigation of plant crops.

The demand for agricultural crops has continued to rise with the fast growth of the population. Extreme climatic changes and environmental stresses constitute a real threat to agricultural food production [16]. Recently, nanobiotechnology has received significant interest in the mitigation of biotic and abiotic stresses [17]. Nanomaterials with different elements have been utilized to improve plant growth and productivity, especially under biotic/abiotic stresses [18]. Among them, silica nanoparticles (SiNPs) have appeared as a critical factor in increasing plant growth and enhancing plant resistance against biotic and abiotic stresses, particularly drought and salinity [19]. The nano-sized form of silica is more readily absorbed by plant roots and/or leaves than the ordinary silica form since the particle size of the SiNPs is below 100 nm [20]. The accumulation of SiNPs in plant leaves increases plant resistance against stress factors such as reactive oxygen species (ROS) and membrane lipid peroxidation (MDA), and activates defensive enzyme activity [21]. SiNPs prevent the admission of Na ions into plants [22]. Hence, foliar spraying of SiNPs may ameliorate plant tolerance of soil salinity and irrigation with saline water [23]. On the other hand, the information on the effects of SiNPs on barley plants under soil salinity and irrigation with saline water is still scarce. 

To increase plant resistance against stress factors such as salt-affected soil and low-quality irrigation water, the application of plant growth-promoting rhizobacteria (PGPR) is an environmentally, economically, and agronomically sound practice, which has already been stated by many authors [24]. It has been demonstrated that seed inoculation with multiple bacterial strains functions better than with a single bacterial strain. The PGPR secretes a considerable amount of plant growth stimulants such as indole acetic acid, gibberellins, and cytokines, and ACC deaminase enzyme (1-aminocyclopropane-1-carboxylate deaminase); at the same time, it inhibits the secretion of plant growth inhibitors such as ethylene, phenols, and abscisic acid (ABA) [25,26]. PGPR improves osmoregulation and activation of antioxidant defense, resulting in higher plant growth and development [27]. Thus, to attain agricultural sustainability, the application of PGPR has gained prime importance in recent years.

The present investigation attempts to provide novel insight into foliar spraying with SiNPs toward abiotic stress tolerance in plants and seed inoculation by multiple bacterial strains. This study will also shed light on the direct impact of the combined application of SiNPs and PGPR on barley growth and productivity irrigated with saline water and grown in salt-affected soil through its influence on physiological and biochemical processes.

## 2. Results

### 2.1. Response of Soil Chemical Properties to the Application of PGPR and SiNPs 

Table 1 illustrates that the utilization of saline water to irrigate barley plants in salt-affected soil increased soil pH compared to freshwater. Nonetheless, the application of PGPR, SiNPs, or their combination considerably reduced soil pH, regardless of the irrigation water type. For instance, the application of PGPR + SiNPs significantly lowered pH from 8.15 (for control) to 8.01. Moreover, the decline in soil pH after treating barley plants with PGPR + SiNPs irrigated with either fresh or saline water was almost the same, recording a reduction of 1.5% and 1.4%, respectively. 

Likewise, the irrigation of barley plants with saline water increased the electrical conductivity of the soil solution (ECe). Yet, the singular and combined exogenous application of PGPR and SiNPs significantly diminished the ECe; however, the PGPR + SiNPs treatment displayed the lowest value of ECe (4.42 dS m^−1^) (Table 1). Furthermore, the reduction in ECe was greater after irrigating barley plants with fresh water rather than saline water. Similar findings and impacts were reported for the content of soil cations, i.e., Na^+^, K^+^, Ca^2+^, and Mg^2+^, in both seasons due to the treatment of barley plants with PGPR, SiNPs, and their combination, regardless of the source of irrigation water (Table 1). Consequently, the ESP of soil displayed the same response. The utilization of saline water in irrigating barley plants in salt-affected soil increased the content of soil cations and ESP compared to fresh water. However, after the singular or combined application of PGPR and SiNPs, the values of Na^+^, SAR, and ESP were significantly reduced while K^+^, Ca^2+^, and Mg^2+^ were considerably increased. The biggest reduction corresponded to the irrigation of barley plants with fresh water. Similar responses were noticed during the two growing seasons (Table 1).

### 2.2. Soil Microbiota and Enzyme Activities

The total count of bacteria, *azotobacter*, and spore-forming bacteria (*Bacillus* spp.) and the activity of soil dehydrogenase and urease were significantly lowered after irrigation of barley plants grown in salt-affected soil with saline water (Table 2). However, in the 2020/2021 season, the total count of soil microbial populations and activity of soil enzymes were higher than in the 2019/2020 season. Overall, the combined application of PGPR and SiNPs (PGPR + SiNPs) to barley plants irrigated with freshwater enhanced the total counts of bacteria, *azotobacter*, and spore-forming bacteria, followed by the sole application of PGPR and SiNPs in both growing seasons. Moreover, the activity of urease (mg TPF g^−1^ soil d^−1^) and dehydrogenase (mg NH4^+^ -N g^−1^ soil d^−1^) enzymes in the soil was significantly (*P*≤ 0.05) decreased due to the irrigation of barley plants with saline water in the salt-affected soil in 2019/2020 and 2020/2021 (Table 2). Nevertheless, the individual and combined application of PGPR and SiNPs appreciably alleviated the injurious effect of saline irrigation water in salt-affected soil on the activity of soil enzymes. For instance, the application of PGPR + SiNPs markedly increased the dehydrogenase activity, regardless of the source of irrigation water. The highest urease activity corresponded to the soil irrigated with fresh water and received the combined application of PGPR and SiNPs. The single application of PGPR displayed higher urease activity than the sole application of SiNPs.

### 2.3. Physiological Responses of Barley Plants Irrigated with Saline Water to PGPR and SiNP Application

#### 2.3.1. Relative Water Content

The relative water content (RWC) in barley leaves was considerably reduced because of the utilization of saline water in irrigating barley plants compared to fresh water during the 2019/2020 and 2020/2021 seasons; however, in the second growing season, this reduction was to a lesser extent (Table 3). The exogenous application of PGPR and SiNPs markedly mitigated the detrimental impact of irrigation with saline water on the RWC of barley leaves in salt-affected soil. Although the single application of PGPR or SiNPs substantially improved the RWC of barley plants, the combined application of PGPR and SiNPs induced an increase in the RWC, recording the highest RWC (81.33%).

#### 2.3.2. Na^+^, K^+^, and K^+^/Na^+^ in Barley Leaves

The accumulation of Na+ in barley leaves significantly increased after irrigation of the plants with saline water compared to the control (fresh water). However, the irrigated plants with saline water during the second growing season showed a lower accumulation rate of Na^+^ in their leaf tissues compared to the first season (Table 3). The exogenous application of PGPR and SiNPs considerably increased the plant resistance to salinity by lowering the uptake and accumulation of Na^+^ in the barley leaves. The treatment of the plants with PGPR + SiNPs exhibited the best effect on the Na^+^ accumulation, displaying the lowest Na+ content in the barley leaves. Changes in the uptake and accumulation of K^+^ in the leaf tissues of barley irrigated with saline water in salt-affected soil were in contrast to those previously reported for Na^+^. Exploiting saline water in the irrigation of barley plants dramatically decreased the K+ content (0.79 and 0.85 mg g^−1^ in the first and second growing seasons, respectively); however, the K^+^ content significantly increased in plants subjected to the combined application of PGPR and SiNPs, which recorded the highest K^+^ content (1.83 mg g^−1^). Consequently, the results of the K^+^/Na^+^ ratio displayed a similar trend to the K^+^ content. The lowered K^+^/Na^+^ ratio (0.52 and 0.62 mg g^−1^) after the irrigation of barley plants with saline water increased after treating plants singularly with PGPR (1.07) and SiNPs (1.29). The K^+^/Na^+^ ratio of treated plants with PGPR + SiNPs was the highest (1.97).

#### 2.3.3. Hydrogen Peroxide and Lipid Peroxidation

The use of saline water in irrigating barley plants in salt-affected soil negatively affected the growth and development of barley plants, recording high contents of hydrogen peroxide (H_2_O_2_) and malondialdehyde (MDA) compared to those plants irrigated with fresh water in both growing seasons (Table 3). In the first growing season, the H_2_O_2_ content increased from 2.34 (fresh water) to 3.03 µmol g^−1^ FW (saline water), regardless of the applied treatments. The application of PGPR, SiNPs, and their combination significantly diminished the content of H_2_O_2_ as they displayed a lower H_2_O_2_ content than the control. The lowest H_2_O_2_ content (1.48 µmol g^−1^ FW) corresponded to the PGPR + SiNPs treatment. The MDA content, as an indicator of the oxidation rate of the lipid bilayer of the cell membrane, significantly increased after irrigation of the plants with saline water in both seasons. For instance, the MDA content increased from 5.51 (fresh water) to 12.47 nmol g^−1^ FW (saline water) during the first season and from 6.50 (fresh water) to 12.97 nmol g^−1^ FW (saline water) within the second season. The lowest MDA content corresponded to the treatment of PGPR + SiNPs (4.99 nmol g^−1^ FW), followed by SiNPs (8.40 nmol g^−1^ FW) and PGPR (10.35 nmol g^−1^ FW), regardless of the growing season.

#### 2.3.4. Relative Chlorophyll Content (SPAD) and Stomatal Conductance 

**Figure 1:**
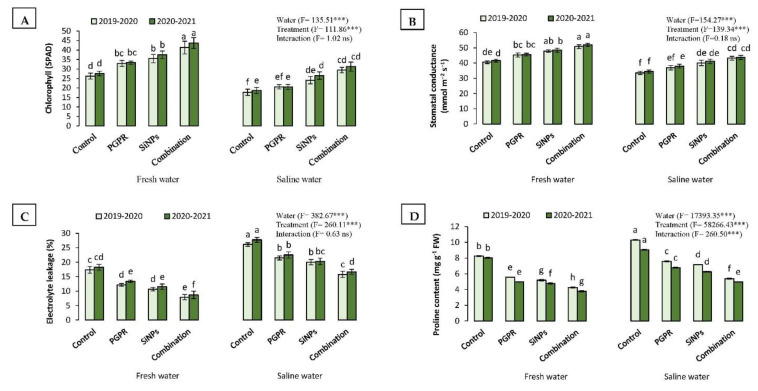
(**A**) Relative chlorophyll content (SPAD); (**B**) stomatal conductance; (**C**) electrolyte leakage; and (**D**) proline content of barley (*Hordum vulgare* L., cv. Giza 132), which was cultivated in salt-affected soil and treated with plant growth-promoting rhizobacteria (PGPR; *Azotobacter chroococcum* SARS 10 and *Pseudomonas koreensis* MG209738), silica nanoparticles (SiNPs), and their combination to alleviate the detrimental impacts of saline irrigation water during two consecutive seasons. Different letters on the same columns of the same season are significant according to the Tukey’s test (*p* ≤ 0.05). Two-way ANOVA was run to display the significant differences between types of irrigation water, treatments, and their interaction according to the Tukey’s test (*p* ≤ 0.05). Data are the means ± SD and *n* = 3. *** denotes significance at *p* ≤ 0.001, ns denotes insignificant difference.

Barley plants irrigated with saline water showed a considerable decrease in the chlorophyll content and stomatal conductance compared to plants irrigated with fresh water. The chlorophyll content dropped from 46.11 (fresh water) to 38.44 SPAD (saline water) in the first season; similar results were also reported in the second season. Nevertheless, the chlorophyll content increased upon treating plants with PGPR and SiNPs, singularly or in combination (Figure 1). The highest chlorophyll content (47.37 SPAD) corresponded to the treatment of PGPR + SiNPs. Similarly, the stomatal conductance decreased from 34.02 (fresh water) to 22.99 mmol m^−2^ s^−1^ (saline water) in the first season and from 35.26 (fresh water) to 24.26 mmol m^−2^ s^−1^ (saline water) in the second season. The single application of PGPR (26.88 mmol m^−2^ s^−1^) and SiNPs (30.91 mmol m^−2^ s^−1^) significantly increased the stomatal conductance compared to untreated plants (22.59 mmol m^−2^ s^−1^); however, the highest stomatal conductance (36.42 mmol m^−2^ s^−1^) corresponded to the combined application of PGPR and SiNPs. 

#### 2.3.5. Electrolyte Leakage (EL) and Proline Content

In contrast to the chlorophyll content and stomatal conductance, barley plants irrigated with saline water exhibited higher electrolyte leakage (EL) and proline content than those watered with fresh water. The EL significantly increased from 12.02% (fresh water) to 20.84% (saline water) in the first season and from 12.96% (fresh water) to 21.79% (saline water) in the second season (Figure 1). The exogenous application of PGPR and SiNPs successfully reduced the EL. The lowest EL (12.25%) corresponded to the treatment of PGPR + SiNPs followed by SiNPs (15.60%) and PGPR (17.38%) while the control plants showed the highest EL (22.38%), regardless of the growing season (Figure 1). Likewise, the proline content in barley leaves irrigated with saline water was higher than in those watered with fresh water in both growing seasons. For example, in the first season, the proline content increased from 5.82 (fresh water) to 7.60 mg g^−1^ FW (saline water). Yet, both the singular and combined application of PGPR and SiNPs revealed a decrease in the proline content compared to the untreated plants (control). The treatment of PGPR + SiNPs showed the lowest proline content (4.60 mg g^−1^ FW), followed by SiNPs (5.84 mg g^−1^ FW) and PGPR (6.22 mg g^−1^ FW).

#### 2.3.6. The Antioxidant Capacity of Barley Plants Irrigated with Saline Water in the Presence of PGPR and SiNPs 

**Figure 2:**
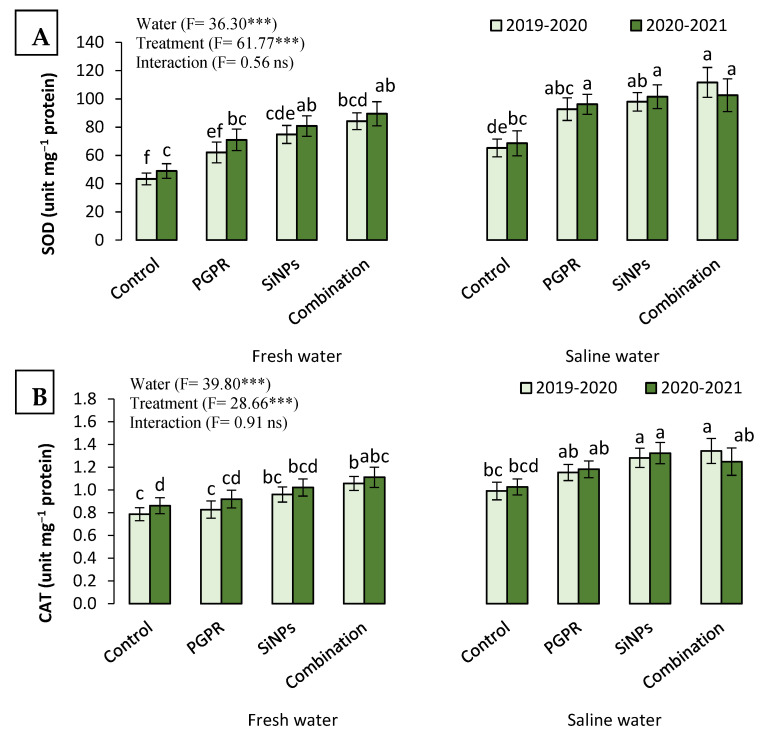

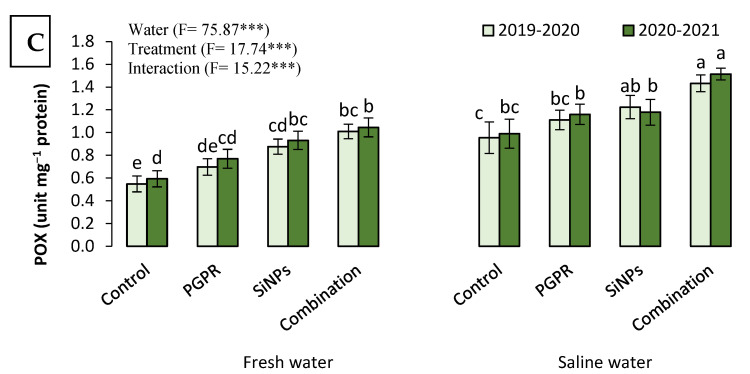
Response of different antioxidant enzymes: (**A**) superoxide dismutase (SOD); (**B**) catalase (CAT); and (**C**) peroxidase (POX) in barley (*Hordum vulgare* L., cv. Giza 132) leaves, which was cultivated in salt-affected soil and treated with plant growth-promoting rhizobacteria (PGPR; *Azotobacter chroococcum* SARS 10 and *Pseudomonas koreensis* MG209738), silica nanoparticles (SiNPs), and their combination to alleviate the detrimental impacts of saline irrigation water during two consecutive seasons. Different letters in the same columns of the same season are significant according to the Tukey’s test (*p* ≤ 0.05). Two-way ANOVA was run to display the significant differences between types of irrigation water, treatments, and their interaction according to the Tukey’s test (*p* ≤ 0.05). Data are the means ± SD and *n* = 3. *** denotes significance at *p* ≤ 0.001, ns denotes insignificant difference.

As a response to salinity stress, the activity of superoxide dismutase (SOD), catalase (CAT), and peroxidase (POX) enzymes increased after the irrigation of barley plants with saline water compared to those watered with fresh water. The SOD activity increased from 66.08 51 (fresh water) to 91.87 51 unit mg^−1^ protein (saline water) in the first growing season and from 72.51 (fresh water) to 92.20 unit mg^−1^ protein (saline water) in the second growing season (Figure 2). The exogenous application of PGPR and SiNPs induced the activity of SOD, recording higher values than untreated plants (control). For instance, the SOD activity of control plants was 56.49 unit mg^−1^ protein and significantly increased to 96.96 unit mg^−1^ protein when plants received the combined application of PGPR and SiNPs. Likely, the CAT activity increased from 0.92 (for control) to 1.19 unit mg^−1^ protein (for plants that received PGPR + SiNPs). Additionally, the exploitation of saline water to irrigate barley plants showed higher CAT activity than those watered with fresh water. Similarly, the POX activity increased after watering plants with saline water compared to fresh water. Moreover, the combined application of PGPR and SiNPs resulted in the highest POX activity (1.11 unit mg^−1^ protein) while plants that received PGPR possessed the lowest POX activity (0.85 unit mg^−1^ protein).

### 2.4. Productivity of Barley Watered with Saline Water in the Presence of PGPR and SiNPs

#### 2.4.1. Yield and Yield-Related Traits of Barley

The spike length, the number of grains per spike, and 1000-grain weight of barley plants appreciably declined due to watering barley with saline water compared to fresh water in both seasons. Yet, the harmful impacts of the irrigation of barley plants with saline water were considerably diminished by treating plants with PGPR, SiNPs, or their mixture. The irrigation of barley plants with saline water in the presence of PGPR + SiNPs exhibited considerable differences in the spike length, number of grains per spike, and 1000-grain weight compared to plants irrigated with fresh water (control). Likewise, the findings stated in Table 4 showed that the largest spike length, number of grains per spike, and 1000-grain weight corresponded to plants treated with the PGPR and SiNPs and irrigated with fresh water through the 2019/2020 and 2020/2021 seasons in salt-affected soil. 

The yield and HI of barley plants was considerably reduced due to the irrigation of the barley crop with saline water relative to fresh water (Table 4). Nevertheless, the harmful effect of irrigation with saline water was appreciably amended by the addition of PGPR and/or SiNPs. Interestingly, the application of PGPR and SiNPs to barley plants under the irrigation condition with saline water significantly increased the grain and straw yields compared to control plants irrigated with freshwater (Table 4). The irrigation of barley plants with fresh water resulted in the highest grain yield, straw yield, and harvest index after the combined application of PGPR and SiNPs through the 2019/2020 and 2020/2021 seasons in salt-affected soil.

#### 2.4.2. N, P, and K Uptake

**Figure 3:**
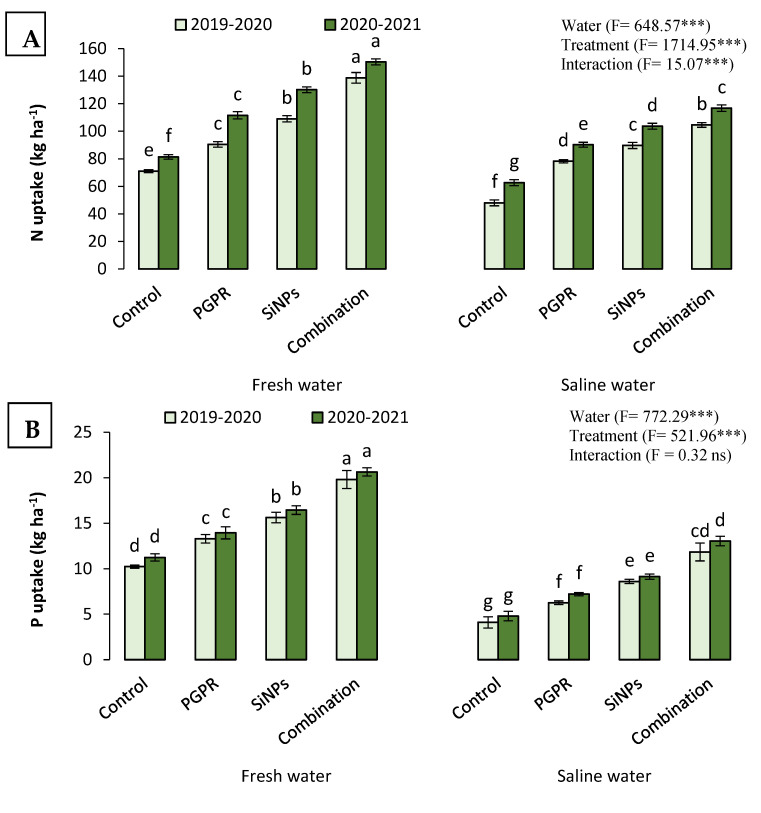

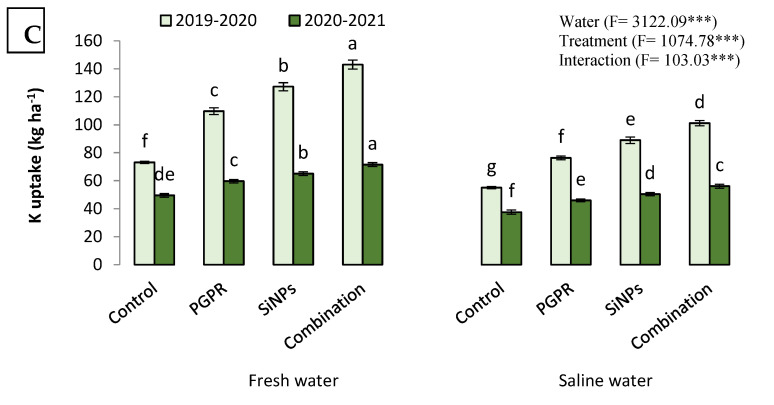
Variations of the uptake of: (**A**) N; (**B**) P; and (**C**) K by barley (*Hordum vulgare* L., cv. Giza 132), which was cultivated in salt-affected soil and treated with plant growth-promoting rhizobacteria (PGPR; *Azotobacter chroococcum* SARS 10 and *Pseudomonas koreensis* MG209738), silica nanoparticles (SiNPs), and their combination to alleviate the detrimental impacts of saline irrigation water during two consecutive seasons. Different letters in the same columns of the same season are significant according to the Tukey’s test (*p* ≤ 0.05). Two-way ANOVA was run to display the significant differences between types of irrigation water, treatments, and their interaction according to the Tukey’s test (*p* ≤ 0.05). Data are the means ± SD and *n* = 3. *** denotes significance at *p* ≤ 0.001, ns denotes insignificant difference.

The irrigation of barley plants with saline water in salt-affected soil considerably decreased the uptake of N, P, and K and their accumulation in barley grains (Figure 3). Nevertheless, the harmful impacts of saline water were considerably mitigated when barley plants were treated with PGPR, SiNPs, or their combined application. Treatment of barley plants with PGPR and SiNPs considerably stimulated the accumulation of N, P, and K in barley grain compared to untreated plants. Moreover, the results depicted in Figure 3 state that the highest N, P, and K uptake corresponded to barley plants treated with PGPR + SiNPs, regardless of the source of irrigation water in both growing seasons.

## 3. Discussion

Overpopulation and climatic changes are forcing us to direct saline soils and low-quality water toward agricultural production. This situation is worse in arid and semi-arid regions. Saline soils and the utilization of low-quality water in crop irrigation are important environmental inhibitors that can influence the growth, morphology, physiology, and production of barley, especially in arid and semi-arid regions [28]. The current investigation aimed to alleviate the detrimental impacts of saline water in the irrigation of barley plants grown in salt-affected soil by the exogenous application of PGPR and SiNPs, added individually or in a combination [29]. The cultivation of crops in saline soils and irrigation with low-quality water may cause an increase in osmotic stress that hinders water uptake, causing a reduction in plant growth. In the present study, the sole application of two bacterial strains (PGPR) significantly reduced the ion toxicity due to the decrease in the concentration of Na^+^ and Cl^-^ in the soil solution while leading to an increase in the contents of K^+^, Ca^2+^, and Mg^2+^ due to the phenomenon of antagonism, which facilitates water uptake in addition to lower pH, EC, SAR, and ESP [30]. Moreover, the application of PGPR increased the excretion of indole acetic acid (IAA) and bacterial exopolysaccharide from soil microbes, which is one of the main steps in the improvement of soil properties. by reducing the concentration of Na^+^, which antagonistically increases the phytoavailability of other nutrients, mainly K^+^, Ca^2+^, and Mg^2+^ [31]. The application of PGPR, directly and indirectly, boosts the growth and development of plants growing under salinity stress conditions. This includes several mechanisms related to the uptake of water and nutrients, photosynthesis pigments, balancing of osmotic equilibrium and ions homeostasis, phytohormone production, up- and-downregulation of different genes, protein synthesis and functions, and salt compartmentalization [32,33].

Additionally, spraying barley plants with SiNPs increased the uptake and accumulation of K^+^ in the leaf tissues while reducing the Na^+^ uptake, which improved the nutrient balance inside the leaves and water uptake [34]. In their study on the effect of different silica sources, i.e., nanosilica, sodium silicate, microsilica, and silicic acid, applied at a rate of 0.5 g kg^-1^ of fine sandy load soil, [35] reported that nanosilica enhanced the growth of soil microbes as reflected in an increased microbial biomass compared to the other applied silica sources. They directed this to the size-dependent traits of nanosilica that facilitate the uptake and building up of silica in the cell of soil microorganisms. They also introduced another hypothesis regarding the positive impacts of nanosilica on the development of soil microorganisms, which is the synergistic uptake of phosphorus on the exogenous application of nanosilica. The authors of [36] cited that the soil application of SiNPs significantly increased the salinity tolerance of cucumber plants, particularly under water deficit conditions. They attributed this improvement of cucumber growth to the elevation of the K/Na ratio due to the application of SiNPs, and thus better root growth induces the release of several root exudates, which act as substrates for soil microorganisms. The combined application of PGPR and SiNPs showed a chelating effect as biostimulators on essential nutrients through their journey from the soil solution to the barley leaves [37]. The application of PGPR increased the numbers of *azotobacter, azospirrillum*, and *bacillus* bacterium in the rhizosphere zone under saline soil and saline water conditions compared to control plants due to an increase in soil enzymes [38]. Urease activity is a soil enzyme that facilitates the breakdown of the urea molecule, transforming it into ammonia, carbon dioxide, and water, leading to high losses of N into the atmosphere [39]. Though the dehydrogenase activity in the soil reflects the total oxidative activity of the microbiota, it can act as a good indicator of the microbial activity in the soil [40]. The higher activities of urease and dehydrogenase increase the phytoavailability of the essential nutrients, physicochemical attributes, and soil health under saline soil and saline water, which improve the root exudates in the rhizosphere and consequently enlarges the microbial community, which is closely associated with the production of phytohormones, including auxins, cytokinins, and gibberellins, and inhibition of the production of ethylene, ABA, and phenols [41].

The present study proved that barley plants irrigated with saline water and grown in salt-affected soil maintained a declined RWC, chlorophyll content (SPAD), and stomatal conductance while oxidative stress indicators such as H_2_O_2_, MDA, EL, and proline content showed an increase [42]. However, the addition of PGPR + SiNPs increased cell elongation and division, resulting in abiotic stress being overcome and an increase in the RWC, chlorophyll content (SPAD), stomatal conductance, and antioxidant enzymes (SOD, CAT, and POX). On the other hand, the same treatment alleviated the salinity stress by reducing the oxidative stress indicators, H_2_O_2_, MDA, EL, and proline content [43]. This improvement in plant health may be due to the reduction in Na^+^ uptake and the elevation in the absorption of K^+^, Ca^2+^, and Mg^2+^, improving the nutrient balance and water uptake by barley plants. The exogenous application of SiNPs increased plant growth and development by expanding the leaf area for higher light interception, causing higher rates of photosynthesis, regulating the stomatal aperture, and improving physiological processes due to the improvement of the leaf water status and osmoregulation and increase in nutrient uptake, which has a substantial role in alleviating the harmful impacts of abiotic stress [44].

In the present study, barley plants irrigated with saline water and grown in salt-affected soil displayed an imbalance between reactive oxygen species (ROS) production and the antioxidant defense system, and thus cellular membrane damage and EL increased. Salt-stressed plants showed an increase in the activity of antioxidant enzymes; however, this increase was not enough to scavenge the ROS. The single application of PGPR and SiNPs caused a noticeable increase in the SOD, CAT, and POX activities [45]. Yet, the combined application of PGPR and SiNPs showed greater potential regarding the activity of antioxidant enzymes, resulting in a further increase in the SOD, CAT, and POX activities. The conversion of H_2_O_2_ into non-toxic compounds (i.e., H_2_O and O_2_) protects the plants against the harmful impacts of H_2_O_2_ on the cell membranes and macromolecules [46,47]. Our results illustrated a distinguished decrease in the H_2_O_2_ contents due to the sole application of PGPR and SiNPs and a reduction in lipid peroxidation (MDA) compared to control plants [48]. In addition, the combined application of PGPR and SiNPs exhibited further alleviation of the oxidative damage compared to the sole application of PGPR and SiNPs due to the transformation of ROS into less or non-toxic compounds [49]. Proline has been depicted as an osmoprotectant that accumulates in response to saline water and saline soil and has a potential role in the defense system of stressed plants through increased enzyme activity and scavenging of free radicals [50].

The utilization of saline water in the irrigation of plant crops, particularly in salt-affected soil, causes an observed decrease in crop productivity, mainly due to the increased oxidative damage. The sole application of PGPR and SiNPs showed a noticeable reduction in the harmful impacts of irrigating barley plants with saline water in salt-affected soil [51]. Yet, the combined application of PGPR and SiNPs further increased their positive effects. The PGPR + SiNPs treatment not only enhanced the plant defense system but also stimulated systemic resistance in barley plants versus the irrigation with saline water in salt-affected soil. PGPR can secrete auxins and ACC deaminase, which play a vital role as growth promoters, leading to an increase in nutrient and water uptake that could enhance physiological processes [52]. These benefits positively induced the grain yield and productivity.

The yield and yield-related traits of barley, including the spike length (cm), number of grains per spike, 1000-grain weight (gm), grain yield, straw yields, biological yield, and HI, were harmfully affected by the utilization of saline water in the irrigation of barley plants in salt-affected soil, owing to the decrease in the assimilation and transfer of the nutrients through grain ripeness and their filling rates [53]. Additionally, saline water and salt-affected soil negatively affected the ovary maturity and seed sterility and consequently resulted in a reduction in yield [54]. The combined application of PGPR and SiNPs effectively enhanced the yield, yield-related traits, and NPK uptake by barley grains [55]. The application of PGPR + SiNPs has the potential to decrease osmotic stress, restrain Na^+^ absorption, and increase K^+^ absorption, resulting in an enhancement of photosynthesis [56]. All of the above-mentioned benefits were further improved by the combined application of PGPR and SiNPs, which exhibited higher seed fertility with lower sterility combined with heavy seed, which caused high yield and nutrient uptake (N, P, and K), under saline irrigation water in salt-affected soil.

Finally, although the exogenous application of SiNPs has several well-documented advantages, especially in boosting plant growth and its productivity under abiotic stresses such as salinity and water deficit and biotic stress such as pests and insects, more investigations on the possible/expected toxicity of the usage of nanoparticles (mainly SiNPs) to plants, the soil ecosystem, animals, and humans are still needed as previously highlighted by [57].

## 4. Materials and Methods

### 4.1. Experimental Layout and Growth Conditions 

A field experiment was conducted to examine the impacts of grain inoculation with plant growth-promoting rhizobacteria (PGPR; *Azotobacter chroococcum* SARS 10 and *Pseudomonas koreensis* MG209738) and foliar spraying with silica nanoparticles (SiNPs) on barley plants (*Hordum vulgare* L., cv. Giza 132) irrigated with saline water in salt-affected soil during the two consecutive winter growing seasons (2019/2020 and 2020/2021) at El-Karada Water Requirements Research Station, Kafr El-Sheikh (North Delta), Egypt (latitude: 31°6’ N/longitude: 30°56′ E). The experiment was arranged in a split-block design with four replications. The types of irrigation water (fresh water and saline water) were allocated in the horizontal (main) plots while amendment treatments (control (untreated plants), PGPR, SiNPs, and PGPR + SiNPs) were allocated in the vertical (submain) plots. The horizontal plots were well separated to avoid infiltration when irrigation was applied. The two bacterial strains were selected according to the experimental laboratory investigations that were carried out previously under different osmotic stresses [58]. The two bacterial strains were obtained from Department of Agricultural Microbiology, Soils, Water, and Environment Research Institute (SWERI), Agricultural Research Centre (ARC), Egypt. Prior to grain sowing, these were inoculated by a mixture (1:1) of the two bacterial strains (prepared by the addition of 15 mL of 10^8^ CFU mL^−1^ from each culture to 30 g of the sterilized carrier) at a rate of 950 g ha^−1^. The SiNPs were obtained from the Faculty of Agriculture, El-Sada Branch, AL-Azhar University, Egypt, and applied as a foliar application. The properties of the used SiNPs were 99.5% purity and 10–20 nm particle size, specific surface area (270–330 m^2^ g^−1^), pH (4.0–4.5), and mean diameter (10 nm). The exogenous foliar application of the SiNPs was applied at a rate of 500 mg SiNPs L^−1^ twice 40 and 60 days after sowing. The weather conditions (Table 5) were attained from the agro-meteorological Sakha Station, Kafrelsheikh Governorate, Egypt located 2 km from the experimental farm. The seeding rate was 120 kg grains ha^−1^, which was provided by the Barley Research Department, Sakha, Kafr El-Sheikh, Egypt. Grains were planted on 25 November during the 2019/2020 season and on 28 November during the 2020/2021 season. Agronomical practices were consistent with the recommendations obtained from the Ministry of Agriculture and Land Reclamation, Egypt as follows: calcium superphosphate (15.5% P_2_O_5_) was added during the seedbed preparation before sowing at a level of 125 kg ha^−1^ and potassium sulfate (48% K_2_O) was broadcasted and incorporated during soil tillage at a rate of 120 kg ha^−1^. Nitrogen fertilizer was added in the form of urea (46% N) at a rate of 288 kg ha^−1^ in two equal doses; the first dose accounted for 40% of the total dose and was applied before the first irrigation while the second dose accounted for 60% and was applied before the second irrigation. 

Soil samples were collected at a depth of 0–30 cm (Table 6) before the planting date of each season by a soil auger and kept in polyethylene bags stored at −20 °C for further analysis.

The characteristics of the irrigation water (Table 7) have been described by the Soil Improvement and Conservation Department, Agricultural Research Center, Giza, Egypt.

### 4.2. Measurements

#### 4.2.1. Soil Analysis

Soil samples were collected from each experimental unit (plot) at the harvest time by an auger. The obtained soil samples were air dried, smashed, passed through a 2 mm sieve, and kept in polyethylene bags for further physicochemical analysis. The soil pH was estimated in a soil:water (1:2.5) suspension using distilled water by a pH meter (Genway, Cole-Parmer, Beacon Road, Stone, Staffordshire, ST15 OSA, UK). The soil electrical conductivity (ECe) (dS m^−1^) was estimated in soil paste extract using an EC meter (Genway, UK). The concentrations of Na^+^, K^+^, Ca^2+^, and Mg^2+^ anions (meq L^−1^) were measured in soil paste extract by an Atomic Absorption Spectrophotometer (AAS, Perkin Elmer 3300, US) with a detection limit of 100 ppb. The exchangeable sodium percentage (ESP) was computed based on the computation of Seilsepour et al. [59]: ESP = 1.95 + 1.03 × SAR (R^2^ = 0.92), where SAR (sodium adsorption ratio) was computed using the computation stated by Richards [60]:

$$SAR=\left[Na^{+}\right]/\sqrt{\frac{\left(\left[Ca^{2}{}^{+}\right]+\left[Mg^{2}{}^{+}\right]\right)}{2}}$$

where Na^+^, Ca^2+^, and Mg^2+^ were expressed in meq L^−1^.

#### 4.2.2. Microbiological Analysis

Eighty days after planting, the total microbial count was determined by the plating method using modified Bunt and Rovira medium and the decimal plate count technique while the total count of *Pseudomonas koreensis* was estimated by King’s B agar medium based on Cochran [61]. The most probable number of *Azotobacter chroococcum* was estimated after incubating the test tubes at 30 ± 2 °C and computed using the tables of Casida et al. [62].

#### 4.2.3. Determination of Soil Enzymes Activity 

Eighty days after planting, the activity of the dehydrogenase and urease enzymes was analyzed. Urease activity was measured according to the quantification of ammonia using the spectrophotometric method at 660 nm as described by Alef and Nannipieri [63]. The dehydrogenase activity was determined according to Mersi [64]. The soil samples were homogenized with INT-solution after incubation of the samples with sucrose (8%) for 3 h at 37 °C. The reduced iodonitro-tetrazolium formazan (INTF) was extracted with dimethyl-formamide and ethanol and estimated photometrically at 464 nm. A UV-160A spectrophotometer (Shimadzu, Ram Ram Ji Trader, Gurgaon, Haryana, Japan) was used in these analyses.

### 4.3. Physiological Characteristics of Barley Plants

#### 4.3.1. Relative Water Content (RWC)

The determination of RWC was carried out according to the method of Barrs and Weatherley [65]. Fresh leaf samples (0.5 g) were weighed (FW) and floated on water until a constant weight was attained in the test tubes to measure the turgid weight (TW), and then oven-dried for 24 h at 80 °C to measure the dry weight (DW). The RWC % was computed as follows:

$$\mathrm{RWC},\;\%=\frac{\left(\mathrm{FW}\;-\;\mathrm{DW}\right)}{\left(\mathrm{TW}\;-\;\mathrm{DW}\right)}\times 100$$

#### 4.3.2. Na^+^ and K^+^ Ions in Leaves 

First, 75 days after sowing, the fourth topmost fully expanded leaves were selected to be oven-dried and digested with 8 mL of digestion mixture HNO_3_:HClO_4_ (3:1 *v*/*v*) according to the technique described by Temmingho and Houba [66] to determine the Na^+^ and K^+^ contents and K^+^/Na^+^ as mg g^−1^ dry weight.

### 4.4. Analysis of Oxidative Stress Indicators 

#### 4.4.1. Hydrogen Peroxide

At 75 days after sowing, the H_2_O_2_ content of leaves was colorimetrically calculated as illustrated by Velikova et al. [67]. Leaf samples were extracted with homogenization of the sample (0.5 g) using liquid N_2_ and trichloroacetic acid (TCA: 0.1%). Extraction was achieved by centrifugation of the mixed sample at 6000 g for 15 min. The intensity of the yellow color of the supernatant was measured at 415 nm by a spectrophotometer. A standard curve was also plotted under standard conditions and the H_2_O_2_ contents were measured as µmol g^−1^ FW. 

#### 4.4.2. Lipid Peroxidation

At 75 days after sowing, the MDA content in the leaves was determined to measure the membrane damage. Therefore, thiobarbituric acid reactive substance (TBARS) was evaluated according to the method explained by Du and Bramlage [68]. Subsamples (500 mg) were homogenized and ground in liquid N2 and hydro-acetone buffer (4:1 *v*/*v*). Then, 20% trichloroacetic acid (TCA) solution and 0.01% butyl hydroxyl toluene (BHT) were added, and samples were incubated at 95 °C. Following incubation, the homogenized sample was subjected to centrifugation at 10,000 g for 10 min. The absorbance was calculated spectrophotometrically at 532 and 600 nm and expressed in nmol g^−1^ FW.

### 4.5. Chlorophyll Content

First, 75 days after grain sowing, the chlorophyll meter (Model: SPAD-502, Minolta Sensing Ltd., Konica Minolta Sensing, Inc. (Headquarters: Sakai, Osaka, Japan) was used to assess the relative chlorophyll content (SPAD) from ten fully expanded uppermost leaves as described by Ling et al. [69]. The SPAD readings were measured from ten plants within each plot and then the values were averaged to obtain a single value for each plant. 

### 4.6. Stomatal Conductance

First, 75 days after grain sowing, stomatal conductance was assessed on 10 fully expanded uppermost leaves from 9:00 to 12:00 am with the AP4 porometer (Headquarters address, Delta-T Devices Ltd,130 Low Road, Burwell, Cambridge, CB25 0EJ, United Kingdom)) from the adaxial and abaxial surfaces. Assessment was carried out on the top leaf and the front (ra) and backside (rb) of the center of the leaf. Total leaf conductance (rl) is 1∕rl = 1∕ra + 1∕rb and expressed as mmol H_2_O m^−2^ s^−1^.

### 4.7. Electrolyte Leakage

First, 75 days after sowing, electrolyte leakage (EL) was assessed in 10 fully expanded uppermost leaves. Ten discs (1 cm^2^) were collected and washed with distilled water. The obtained samples were placed in test tubes with 10 mL of distilled water. The percentage of EL was measured by the following formula [70]:

$$EL=\frac{\left(C1\right)}{\left(C2\right)}\times 100$$

where C1: EC of samples placed in a water bath for 40 °C for 30 min; C2: EC of the same samples placed in a water bath for 10 min at 100 °C.

### 4.8. Proline Content

Leaf proline content (mg 100 g^−1^ FW) was assessed according to Bates [71]. First, 10 fully expanded uppermost leaves were extracted by 0.5 g of young leaves with sulfuric acid (3%), and the solution was quantified by ninhydrin reagent. The solution was then homogenized with toluene, and the absorbance 500 was assessed by the same spectrophotometer at 520 nm.

### 4.9. Antioxidant System

The antioxidant enzyme extracts were obtained by crushing a 1 g leaf sample in liquid N_2_ and homogenizing it in 5 mL of cold phosphate buffer (50 mM phosphate buffer pH 7.0, containing 1 mM EDTA, 1 mM phenylmethylsulfonyl fluoride, and 1% polyvinylpolypirrolidone) for use as an enzyme extract. Next, the mixed sample was centrifuged at 15,000× *g* at 4 °C for 30 min. The activity of SOD (EC: 1.15.1.1) was assessed according to the 50% NBT reduction assay at 560 nm as explained by Beauchamp and Fridovich [72]. The activity of CAT (EC: 1.11.1.6) was assessed by observing the reaction between 50 µL of enzyme extract and 12.5 mm of H_2_O_2_ in 50 mM K-phosphate buffer (pH 7.0). The reaction started by applying H_2_O_2_ and the absorbance was examined at 240 nm for 60 s as explained by Aebi [73]. The POX (EC: 1.11.1.7) activity was assessed by o-phenylenediamine as a chromogenic marker in the presence of H_2_O_2_ and enzyme extract at 417 nm as explained by Vetter et al. [74]. The activity of all enzymes was expressed as unit mg^−1^ protein.

### 4.10. Crop Yield

At physiological maturity, ten barley plants from each experimental plot were cut using a hand sickle above the soil surface to measure the spike length (cm), number of grains per spike, and 1000-grain weight (g). In addition, six m2 areas were harvested from each experimental unit to determine the grain and straw yield when the moisture content of the grain was below 15%. The biological yield was computed by straw. The harvest index (HI; %) was computed using the following equation:

$$\mathrm{HI},\;\%=\frac{\mathrm{Grain}\;\mathrm{yield}\;\left({t\;\mathrm{ha}}^{-1}\right)}{\mathrm{Biological}\;\mathrm{yield}\;\left({t\;\mathrm{ha}}^{-1}\right)}\times 100$$

### 4.11. Nutrient Uptake

At physiological maturity, air-dried grain samples were placed into a forced-air oven for 48 h at 70 °C. Then, the grain samples were assembled, air-dried, crushed, and prepared for laboratory determination of grain N, P, and K uptake. The N, P, and K contents in the barley grain were assessed by micro Kjeldahl’s according to the method of A.O.A.C. [75] using a spectrophotometer and flame photometer according to the method of Sparks et al. [76], respectively.

### 4.12. Statistical Analysis

The normality and homoscedasticity of the dependent variables were checked and transformed as necessary. Data analysis was performed using Microsoft Excel 2016 and the SPSS 25.0 software package (SPSS Inc., Chicago, IL, USA). The analysis of variance using one-way ANOVA was performed separately between treatments, seasons, and water types. Separation of the means was performed by the post-hoc test (Tukey’s test), and significant differences were accepted at the level *p* ≤ 0.05. The analysis of variance using two-way ANOVA was performed between water types, seasons, and treatments. The data were presented as the mean ± standard deviation.

## 5. Conclusions

Our findings propose that the synergistic application of PGPR and SiNPs represent a good strategy and beneficial tool to allow the exploitation of low-quality water to filed crops in salt-affected soil, particularly in arid and semiarid zones, as it noticeably mitigates the negative effects of saline water in saline soil. The synergistic application of PGPR and SiNPs reduced soil salinity, resulting in stimulation of the physiological processes and herein enzymatic activity and eventually the barley yield. The soil health, plant development, grain yield, and nutrient uptake of barley improved significantly under irrigation with saline water in salt-affected soil in the presence of seed inoculation with PGPR and foliar spraying of SiNPs. The collaborative impact of PGPR and SiNPs was revealed to be efficient in the mitigation of soil ESP by reducing the content of Na^+^ and oxidative stress. PGPR and SiNPs represent a very promising strategy for improving cereal growth and productivity under saline conditions.

## Figures and Tables

**Table 1:** Alteration of some chemical properties of salt-affected soil cultivated with barley (*Hordum vulgare* L., cv. Giza 132), which was treated with plant growth-promoting rhizobacteria (PGPR; *Azotobacter chroococcum* SARS 10 and *Pseudomonas koreensis* MG209738), silica nanoparticles (SiNPs), and their combination to alleviate the detrimental impacts of saline irrigation water during two consecutive seasons.

			pH *	EC_e_ (dS m^−1^) ^‡^	SAR ^¥^	ESP (%) ^†^
**Water type**						
Fresh water_2019–2020			8.06 ± 0.05 c	4.54 ± 0.68 c	7.16 ± 1.48 c	15.88 ± 2.80 c
Saline water_2019–2020			8.13 ± 0.05 a	5.71 ± 0.38 a	9.69 ± 1.05 a	20.91 ± 2.14 a
Fresh water_2020–2021			8.02 ± 0.06 d	4.12 ± 0.79 d	6.54 ± 1.43 d	14.50 ± 2.96 d
Saline water_2020–2021			8.11 ± 0.07 b	5.38 ± 0.51 b	8.80 ± 1.11 b	18.80 ± 1.99 b
**Treatment**						
Control			8.15 ± 0.05 a	5.77 ± 0.45 a	9.65 ± 1.12 a	20.71 ± 2.27 a
PGPR			8.09 ± 0.00 b	4.88 ± 0.80 b	8.26 ± 1.50 b	17.78 ± 2.80 b
SiNPs			8.07 ± 0.05 c	4.69 ± 0.84 c	7.64 ± 1.64 c	16.75 ± 3.41 c
Combination			8.01 ± 0.06 d	4.42 ± 0.83 d	6.64 ± 1.58 d	14.85 ± 3.14 d
**Interaction**						
Year	Water type	Treatment				
2019–2020	Fresh water	Control	8.11 ± 0.01 c BC	5.50 ± 0.05 c C	9.15 ± 0.48 bc BC	19.82 ± 0.23 c CD
		PGPR	8.06 ± 0.01 de EF	4.52 ± 0.07 e F	7.26 ± 0.32 d E	15.72 ± 0.15 e GH
		SiNPs	8.05 ± 0.01 e FG	4.18 ± 0.034 f G	6.52 ± 0.46 de EFG	14.61 ± 0.10 f I
		Combination	7.99 ± 0.01 f I	3.97 ± 0.03 g H	5.70 ± 0.30 e GH	13.35 ± 0.20 g J
	Saline water	Control	8.19 ± 0.01 a A	6.18 ± 0.04 a A	10.96 ± 0.38 a A	23.61 ± 0.42 a A
		PGPR	8.13 ± 0.01 b B	5.78 ± 0.04 b B	9.96 ± 0.44 ab AB	20.99 ± 0.39 b B
		SiNPs	8.13 ± 0.01 bc B	5.60 ± 0.03 c C	9.36 ± 0.36 bc BC	20.63 ± 0.21 b BC
		Combination	8.08 ± 0.01 d DE	5.27 ± 0.06 d D	8.46 ± 0.43 c CD	18.40 ± 0.32 d EF
2020–2021	Fresh water	Control	8.09 ± 0.01 bc CD	5.28 ± 0.04 bc D	8.38 ± 0.35 b CD	18.25 ± 0.35 c F
		PGPR	8.03 ± 0.01 d GH	3.93 ± 0.04 e H	6.81 ± 0.43 cd EF	15.19 ± 0.18 e HI
		SiNPs	8.01 ± 0.01 d HI	3.79 ± 0.07 f I	5.99 ± 0.35 d FGH	13.25 ± 0.24 f J
		Combination	7.94 ± 0.01 e j	3.49 ± 0.07 g J	5.00 ± 0.28 e H	11.31 ± 0.40 g K
	Saline water	Control	8.19 ± 0.01 a A	6.12 ± 0.01 a A	10.09 ± 0.36 a AB	21.15 ± 0.22 a B
		PGPR	8.11 ± 0.01 b BC	5.30 ± 0.06 b D	9.03 ± 0.24 b BC	19.22 ± 0.28 b DE
		SiNPs	8.09 ± 0.01 c D	5.17 ± 0.03 c D	8.69 ± 0.38 b C	18.52 ± 0.28 bc EF
		Combination	8.03 ± 0.01 d H	4.94 ± 0.02 d E	7.39 ± 0.00 c DE	16.32 ± 0.00 d G
Two-way ANOVA						
Water type			***	***	***	***
Treatment			***	***	***	***
Interaction			***	***	ns	***

* pH is measured in soil:water (1:2.5) suspension; ^‡^ Electrical conductivity is measured in soil paste extract; ^¥^ Sodium adsorption ratio; ^†^ Exchangeable sodium percentage means in the same column within the same season followed by different lowercase letters are significant according to the Tukey’s test (*p* *≤* 0.05) and different uppercase letters are significant regardless of the growing season according to the Tukey’s test (*p* ≤ 0.05). Data are the means ± SD and *n* = 3. *** denotes significance at *p* ≤ 0.001, ns denotes insignificant difference.

**Table 2:** Variation of the total count of some soil microbial populations and enzyme activity of salt-affected soil cultivated with barley (*Hordum vulgare* L., cv. Giza 132), which was treated with plant growth-promoting rhizobacteria (PGPR; *Azotobacter chroococcum* SARS 10 and *Pseudomonas koreensis* MG209738), silica nanoparticles (SiNPs), and their combination to alleviate the detrimental impacts of saline irrigation water during two consecutive seasons.

			Total Microbial Count (Log cfu g^−1^ Soil)			Soil Enzymes	
			Bacteria (×10^7^)	Azotobacter (×10^6^)	*Bacillus* spp. (×10^5^)	Urease (mg NH_4_^+^ g^−1^ dry soil d^−1^)	Dehydrogenase (mg TPF g^−1^ dry soil d^−1^)
**Water type**							
Fresh water_2019–2020			3.72 ± 1.37 b	1.45 ± 0.51 a	2.81 ± 0.88 a	163.2 ± 41.5 b	98.7 ± 36.2 b
Saline water_2019–2020			2.39 ± 0.72 d	1.08 ± 0.51 d	1.64 ± 0.76 d	117.7 ± 28.2 d	73.9 ± 33.1 d
Fresh water_2020–2021			4.75 ± 1.25 a	1.27 ± 0.54 b	2.51 ± 0.92 b	174.0 ± 42.0 a	104.2 ± 38.6 a
Saline water_2020–2021			3.52 ± 1.21 c	1.11 ± 0.52 c	1.72 ± 0.77 c	124.4 ± 27.0 c	82.6 ± 29.5 c
**Treatment**							
Control			2.40 ± 0.80 d	0.59 ± 0.17 d	1.21 ± 0.40 d	106.0 ± 18.0 d	49.4 ± 11.6 d
PGPR			3.74 ± 1.34 b	1.35 ± 0.19 b	2.33 ± 0.83 b	149.0 ± 32.0 b	97.7 ± 14.7 b
SiNPs			3.26 ± 0.70 c	1.15 ± 0.28 c	2.01 ± 0.71 c	135.0 ± 25.0 c	80.8 ± 11.5 c
Combination			4.98 ± 1.27 a	1.82 ± 0.15 a	3.13 ± 0.49 a	189.2 ± 37.0 a	131.6 ± 19.0 a
**Interaction**							
Year	Water type	Treatment					
2019–2020	Fresh water	Control	2.66 ± 0.05 c G	0.73 ± 0.02 f H	1.54 ± 0.02 d FG	116.9 ± 2.39 de GH	56.7 ± 3.06 g H
		PGPR	3.25 ± 0.03 b EF	1.58 ± 0.01 c C	3.14 ± 0.03 b B	168.7 ± 1.84 b D	107.5 ± 2.11 c D
		SiNPs	3.23 ± 0.04 b EF	1.56 ± 0.02 c C	3.04 ± 0.09 b BC	150.7 ± 3.74 c F	87.7 ± 1.15 d E
		Combination	5.74 ± 0.10 a AB	1.94 ± 0.02 a A	3.53 ± 0.04 a A	216.4 ± 2.41 a B	143.0 ± 4.07 a B
	Saline water	Control	1.41 ± 0.05 e I	0.44 ± 0.03 g I	0.84 ± 0.01 e H	86.6 ± 1.74 f J	34.4 ± 2.04 h J
		PGPR	2.61 ± 0.12 c G	1.18 ± 0.01 d E	1.56 ± 0.04 d FG	119.9 ± 3.42 d G	80.6 ± 0.74 e F
		SiNPs	2.40 ± 0.04 d H	1.04 ± 0.02 e F	1.48 ± 0.02 d G	110.0 ± 2.18 e H	66.5 ± 0.77 f G
		Combination	3.14 ± 0.08 b F	1.67 ± 0.03 b B	2.67 ± 0.04 c D	154.4 ± 2.37 c EF	114.1 ± 1.85 b C
2020–2021	Fresh water	Control	3.31 ± 0.05 e EF	0.75 ± 0.01 g H	1.56 ± 0.02 e FG	125.1 ± 3.60 d G	60.3 ± 1.52 e GH
		PGPR	5.69 ± 0.07 b B	1.45 ± 0.01 c D	2.95 ± 0.03 b C	183.6 ± 2.16 b C	112.2 ± 1.79 b CD
		SiNPs	4.10 ± 0.06 d D	0.93 ± 0.01 f G	1.96 ± 0.10 d E	161.5 ± 2.65 c DE	92.1 ± 1.60 c E
		Combination	5.89 ± 0.07 a A	1.95 ± 0.01 a A	3.57 ± 0.04 a A	225.6 ± 3.51 a A	152.4 ± 3.34 a A
	Saline water	Control	2.22 ± 0.04 f H	0.45 ± 0.02 h I	0.89 ± 0.02 f H	95.5 ± 2.10 e I	46.2 ± 2.41 f I
		PGPR	3.41 ± 0.05 e E	1.21 ± 0.01 d E	1.67 ± 0.03 e F	123.9 ± 2.66 d G	90.5 ± 1.64 c E
		SiNPs	3.32 ± 0.05 e EF	1.05 ± 0.02 e F	1.57 ± 0.02 e FG	117.6 ± 3.57 d GH	76.9 ± 2.65 d F
		Combination	5.14 ± 0.05 c C	1.72 ± 0.03 b B	2.76 ± 0.04 c D	160.5 ± 1.61 c DE	117.0 ± 1.42 b C
Two-way ANOVA							
Water type			***	***	***	***	***
Treatment			***	***	***	***	***
Interaction			***	***	***	***	***

Means in the same column within the same season followed by different lowercase letters are significant according to the Tukey’s test (*p* *≤* 0.05) and different uppercase letters are significant regardless of the growing season according to the Tukey’s test (*p* ≤ 0.05). Data are the means ± SD and *n* = 3. *** denotes significance at *p* ≤ 0.001.

**Table 3:** Physiological response of barley (*Hordum vulgare* L., cv. Giza 132) plants cultivated in salt-affected soil to the exogenous application of plant growth-promoting rhizobacteria (PGPR; *Azotobacter chroococcum* SARS 10 and *Pseudomonas koreensis* MG209738), silica nanoparticles (SiNPs), and their combination to alleviate the detrimental impacts of saline irrigation water during two consecutive seasons.

			RWC ^†^ (%)	K/Na Ratio	H_2_O_2_ (µmol g^−1^ FW)	MDA ^‡^ (nmol g^−1^ FW)
**Water type**						
Fresh water_2019–2020			81.42 ± 6.22 b	1.61 ± 0.88 b	2.34 ± 0.92 b	5.51 ± 2.87 b
Saline water_2019–2020			62.19 ± 9.62 d	0.52 ± 0.37 d	3.03 ± 1.07 a	12.47 ± 4.05 a
Fresh water_2020–2021			83.57 ± 7.71 a	1.85 ± 1.08 a	3.22 ± 1.28 a	6.50 ± 2.84 b
Saline water_2020–2021			65.61 ± 10.50 c	0.62 ± 0.46 c	3.32 ± 1.40 a	12.97 ± 4.95 a
**Treatment**						
Control			61.83 ± 11.89 d	0.29 ± 0.20 d	4.26 ± 0.54 a	13.71 ± 4.79 a
PGPR			73.50 ± 13.80 c	1.07 ± 0.70 c	3.27 ± 0.60 b	10.35 ± 4.20 b
SiNPs			76.13 ± 10.97 b	1.29 ± 0.79 b	2.89 ± 0.57 b	8.40 ± 4.33 c
Combination			81.33 ± 6.96 a	1.97 ± 1.01 a	1.48 ± 0.13 c	4.99 ± 2.45 d
**Interaction**						
Year	Water type	Treatment				
2019–2020	Fresh water	Control	72.2 ± 1.70 b CD	0.48 ± 0.02 d FG	3.58 ± 0.46 ab ABCD	9.1 ± 1.1 cd DEFG
		PGPR	83.9 ± 1.47 a A	1.57 ± 0.12 b D	2.34 ± 0.39 bcde CDEFG	6.2 ± 1.8 def FGHI
		SiNPs	84.0 ± 0.70 a A	1.80 ± 0.11 b D	2.10 ± 0.31 cde DEFG	4.2 ± 1.1 ef HI
		Combination	85.6 ± 1.22 a A	2.61 ± 0.22 a B	1.35 ± 0.25 e G	2.4 ± 0.2 f I
	Saline water	Control	50.9 ± 2.77 e G	0.13 ± 0.02 e H	4.19 ± 0.85 a AB	17.4 ± 1.5 a AB
		PGPR	58.9 ± 2.50 d F	0.39 ± 0.05 de G	3.37 ± 0.40 abc BCD	13.2 ± 0.20 b BCD
		SiNPs	65.4 ± 1.86 c E	0.56 ± 0.02 d FG	2.91 ± 0.51 abcd BCDEF	11.6 ± 0.4 bc CDE
		Combination	73.5 ± 1.80 b BC	1.01 ± 0.09 c E	1.64 ± 0.67 de EFG	7.7 ± 1.9 cde EFGH
2020–2021	Fresh water	Control	72.1 ± 1.66 c CD	0.45 ± 0.02 f FG	4.41 ± 0.56 ab AB	10.0 ± 0.0 cd DEF
		PGPR	86.6 ± 1.25 a A	1.79 ± 0.10 c D	3.64 ± 0.49 ab ABC	7.4 ± 1.8 de EFGHI
		SiNPs	87.1 ± 0.35 a A	2.11 ± 0.03 b C	3.43 ± 0.29 b ABCD	5.1 ± 1.7 e GHI
		Combination	88.6 ± 0.70 a A	3.06 ± 0.08 a A	1.40 ± 0.59 c G	3.5 ± 0.5 e HI
	Saline water	Control	52.2 ± 2.04 f G	0.10 ± 0.01 g H	4.88 ± 0.37 a A	18.2 ± 2.4 a A
		PGPR	64.6 ± 1.26 e E	0.52 ± 0.04 ef FG	3.74 ± 0.67 ab ABC	14.6 ± 0.7 ab ABC
		SiNPs	68.1 ± 0.20 d DE	0.67 ± 0.02 e F	3.13 ± 0.36 b BCDE	12.7 ± 1.6 bc CD
		Combination	77.6 ± 0.76 b B	1.21 ± 0.09 d E	1.53 ± 0.38 c FG	6.4 ± 1.1 de FGHI
Two-way ANOVA						
Water type			***	***	***	***
Treatment			***	***	***	***
Interaction			***	***	ns	ns

^†^ Relative water content; ^‡^ malondialdehyde content (lipid peroxidation). Means in the same column within the same season followed by the different lowercase letters are significant according to the Tukey’s test (*p* *≤* 0.05) and different uppercase letters are significant regardless of the growing season according to the Tukey’s test (*p* ≤ 0.05). Data are the means ± SD and *n* = 3. *** denotes significance at *p* ≤ 0.001, ns denotes insignificant difference.

**Table 4:** Yield and yield-related traits of barley (*Hordum vulgare* L., cv. Giza 132), which was cultivated in salt-affected soil and treated with plant growth-promoting rhizobacteria (PGPR; Azotobacter chroococcum SARS 10 and Pseudomonas koreensis MG209738), silica nanoparticles (SiNPs), and their combination to alleviate the detrimental impacts of saline irrigation water during two consecutive seasons.

			Spike Length (cm)	Grain Per Spike	1000-Grain Weight (g)	Grain Yield (kg ha^−1^)	Straw Yield (kg ha^−1^)	Biological Yield (kg ha^−1^)	HI ^†^ (%)
**Water type**									
Fresh water_2019–2020			8.25 ± 0.87 b	54.89 ± 4.98 b	50.07 ± 4.37 b	3188 ± 410 a	5447 ± 101 a	8634 ± 501 b	36.8 ± 2.69 a
Saline water_2019–2020			6.79 ± 0.75 d	46.44 ± 5.07 d	43.66 ± 5.94 d	2599 ± 395 b	5007 ± 439 b	7606 ± 831 c	34.1 ± 1.55 c
Fresh water_2020–2021			8.38 ± 0.91 a	56.47 ± 5.49 a	51.67 ± 5.07 a	3232 ± 351 a	5542 ± 158 a	8774 ± 502 a	36.8 ± 1.95 a
Saline water_2020–2021			6.93 ± 0.79 c	47.75 ± 4.64 c	44.92 ± 3.56 c	2624 ± 355 b	4783 ± 222 c	7406 ± 572 d	35.3 ± 2.13 b
**Treatment**									
Control			6.54 ± 0.63 d	45.27 ± 4.99 d	42.59 ± 3.36 d	2430 ± 335 d	4877 ± 505 c	7306 ± 837 d	33.2 ± 0.84 d
PGPR			7.48 ± 1.00 c	50.12 ± 4.50 c	46.48 ± 3.80 c	2836 ± 379 c	5224 ± 378 b	8060 ± 750 c	35.1 ± 1.55 c
SiNPs			7.82 ± 0.96 b	52.87 ± 5.21 b	48.53 ± 3.60 b	3055 ± 328 b	5266 ± 325 b	8322 ± 636 b	36.7 ± 1.38 b
Combination			8.51 ± 0.78 a	57.29 ± 5.42 a	52.73 ± 4.82 a	3321 ± 346 a	5411 ± 296 a	8732 ± 605 a	38.0 ± 1.68 a
**Interaction**									
Year	Water type	Treatment							
2019–2020	Fresh water	Control	7.03 ± 0.02 d E	48.9 ± 0.46 d HI	44.77 ± 0.38 d HI	2644 ± 39 de EF	5296 ± 89 ab BCDE	7939 ± 85 d E	33.3 ± 0.58 d EF
		PGPR	8.31 ± 0.03 b C	53.3 ± 0.48 c EF	49.22 ± 0.53 c EF	3161 ± 67 bc CD	5499 ± 81 a ABC	8660 ± 124 bc CD	36.5 ± 0.48 bc BCD
		SiNPs	8.55 ± 0.12 b BC	56.7 ± 0.41 b CD	50.96 ± 0.56 b D	3324 ± 70 b BC	5492 ± 102 a ABC	8816 ± 35 b C	37.7 ± 0.93 ab ABC
		Combination	9.09 ± 0.03 a A	60.6 ± 1.09 a B	55.32 ± 0.39 a B	3622 ± 90 a A	5500 ± 226 a ABC	9122 ± 145 a AB	39.7 ± 1.57 a A
	Saline water	Control	5.95 ± 0.07 f H	40.0 ± 1.62 f K	38.86 ± 0.56 f L	2115 ± 45 f G	4407 ± 78 c G	6522 ± 114 f G	32.4 ± 0.34 d F
		PGPR	6.48 ± 0.13 e G	45.6 ± 0.1.42 e J	42.58 ± 0.42 e J	2473 ± 36 e F	4989 ± 23 b EF	7462 ± 44 e F	33.1 ± 0.33 d F
		SiNPs	6.98 ± 0.09 d EF	48.2 ± 0.59 de HI	45.03 ± 0.26 d GHI	2774 ± 71 d E	5199 ± 155 ab CDE	7973 ± 114 d E	34.8 ± 1.18 cd DEF
		Combination	7.73 ± 0.16 c D	52.0 ± 0.69 c FG	48.19 ± 0.48 c F	3033 ± 52 c D	5434 ± 136 a ABC	8467 ± 118 c D	35.8 ± 0.85 bc CDE
2020–2021	Fresh water	Control	7.14 ± 0.05 e E	50.1 ± 0.43 d GH	46.00 ± 0.24 e G	2785 ± 60 d E	5331 ± 114 b BCD	8116 ± 76 c E	34.3 ± 0.91 cd DEF
		PGPR	8.39 ± 0.15 c C	54.7 ± 0.60 c DE	50.24 ± 0.11 c DE	3166 ± 80 c CD	5591 ± 133 ab AB	8757 ± 53 b C	36.2 ± 1.14 bc CD
		SiNPs	8.74 ± 0.06 b B	58.0 ± 0.51 b C	52.23 ± 0.30 b C	3355 ± 49 b B	5540 ± 133 ab AB	8896 ± 86 b BC	37.7 ± 0.90 ab ABC
		Combination	9.27 ± 0.05 a A	63.1 ± 0.74 a A	58.20 ± 0.55 a A	3620 ± 35 a A	5708 ± 54 a A	9328 ± 56 a A	38.8 ± 0.37 a AB
	Saline water	Control	6.04 ± 0.08 g H	42.1 ± 0.56 f K	40.71 ± 0.44 g K	2174 ± 77 f G	4474 ± 67 d G	6648 ± 142 f G	32.7 ± 0.48 d F
		PGPR	6.74 ± 0.14 f FG	46.9 ± 0.72 e IJ	43.88 ± 0.20 f I	2544 ± 50 e F	4819 ± 100 c F	7363 ± 56 e F	34.6 ± 0.90 cd DEF
		SiNPs	6.99 ± 0.08 ef EF	48.6 ± 0.70 de HI	45.90 ± 0.11 e GH	2768 ± 57 d E	4834 ± 88 c F	7602 ± 75 d F	36.4 ± 0.79 bc BCD
		Combination	7.95 ± 0.08 d D	53.4 ± 0.82 c EF	49.19 ± 0.34 d EF	3009 ± 68 c D	5003 ± 15 c DEF	8012 ± 55 c E	37.6 ± 0.59 ab ABC
Two-way ANOVA									
Water type			***	***	***	***	***	***	***
Treatment			***	***	***	***	***	***	***
Interaction			***	ns	***	ns	***	***	ns

**^†^** Harvest index. Means in the same column within the same season followed by different lowercase letters are significant according to the Tukey’s test (*p* *≤* 0.05) and different uppercase letters are significant regardless of the growing season according to the Tukey’s test (*p* ≤ 0.05). Data are the means ± SD and *n* = 3. *** denotes significance at *p* ≤ 0.001, ns denotes insignificant difference.

**Table 5:** Meteorological data of the experimental sites during the 2019/2020 and 2020/2021 growing seasons.

Year Month	2019/2020				2020/2021			
	Temperature (°C)		Rainfall (mm)	Relative Humidity (%)	Temperature (°C)		Rainfall (mm)	Relative Humidity (%)
	Max	Min			Max	Min		
November	27.8	19.6	0.87	34.3	24.3	15.2	0.88	32.6
December	26.7	18.7	0.76	35.4	23.9	16.3	0.99	33.2
January	25.6	16.6	1.65	32.6	22.2	14.4	0.76	34.7
February	23.4	15.4	3.54	34.7	21.3	10.1	3.08	45.4
March	22.3	10.3	6.43	45.8	21.6	11.7	6.35	45.1
April	24.1	11.2	0.57	46.9	23.5	11.5	0.57	43.8

**Table 6:** Physicochemical characteristics of the experimental soil (0–30 cm depth) in the two growing seasons 2018 and 2019.

Character	2019/20	2020/21
pH (1:2.5 soil:water suspension)	8.14 ± 0.01 ^†^	8.15 ± 0.02
Electrical conductivity (EC, dS m^−1^) ^¥^	5.23 ± 0.02	5.34 ± 0.01
Soil organic matter (g kg^−1^)	11.33 ± 0.02	11.4 ± 0.04
ESP ^#^ (%)	22.12 ± 0.21	21.34 ± 0.22
Particle size distribution (%)		
Sand	27.22 ± 1.34	27.22 ± 1.12
Silt	25.23 ± 2.01	25.32 ± 1.44
Clay	47.55 ± 2.34	47.13 ± 2.12
Texture grade	clayey	clayey
Soluble cations (meq L^−1^) ^¥^		
Ca^++^	7.43 ± 0.65	9.12 ± 0.54
Mg^++^	5.23 ± 1.33	6.32 ± 1.32
Na^+^	26.23 ± 2.12	22.43 ± 3.54
K^+^	0.54 ± 0.12	0.44 ± 0.12
Soluble anions (meq L^−1^) ^¥^		
CO_3_^− −^	nd ^‡^	nd
HCO_3_^−^	4.22 ± 0.54	3.65 ± 0.43
Cl^−^	24.33 ± 1.21	18.43 ± 1.12
SO_4_^− −^	15.43 ± 3.45	11.21 ± 3.22
Available macronutrients (mg kg^−1^)		
N	9.43± 0.54	10.12 ± 1.54
P	8.12 ± 1.32	8.23 ± 1.32
K	354 ± 26.12	367 ± 24.12
Total counts of soil microbes		
Bacteria (CFU ×10^7^ g^−1^ dry soil)	32 ± 1.34	37 ± 1.45
Fungi (CFU ×10^4^ g^−1^ dry soil)	11 ± 0.45	16 ± 1.76
Actinomycetes (CFU ×10^5^ g^−1^ dry soil)	22 ± 1.76	25 ± 1.23

^†^ Standard deviation; ^‡^ not detected; ^¥^ measured in soil paste extract; ^#^ exchangeable sodium percentage.

**Table 7:** Characterization of irrigation water during the 2019/2020 and 2020/2021 growing seasons.

Character	Fresh Water		Saline Water *	
	2018	2019	2018	2019
pH	7.32 ± 0.65	7.24 ± 0.66	8.45 ± 0.16	8.26 ± 0.12
EC (dS m^−1^)	0.45 ± 0.12	0.49 ± 0.01	3.87 ± 0.05	3.73 ± 0.11
SAR	1.36 ± 0.12	1.41 ± 0.06	7.65 ± 0.33	7.66 ± 0.22
Na^+^ (mq L^−1^)	1.78 ± 0.13	1.91 ± 0.04	16.54 ± 1.45	16.75 ± 1.21
Cl^−^ (mq L^−1^)	3.47 ± 0.02	3.37 ± 0.05	11.33 ± 0.76	11.75 ± 0.43
SO_4_^−^ (mq L^−1^)	0.25 ± 0.03	0.13 ± 0.02	7.87 ± 0.24	8.44 ± 0.05
NH_4_^+^ (mq L^−1^)	1.36 ± 0.04	1.82 ± 0.04	2.16 ± 0.05	2.33 ± 0.03
COD (mq L^−1^)	12.45 ± 0.88	11.11 ± 1.06	nd ^¥^	nd
BOD (mq L^−1^)	5.36 ± 0.27	5.24 ± 0.87	nd	nd
SS (mq L^−1^)	174 ± 12.48	183 ± 13.4	18 ± 1.3	15 ± 1.4
DS (mq L^−1^)	365 ± 33	387 ± 36	2976 ± 154	2944 ± 123

COD: chemical oxygen demand; BOD: biological oxygen demand; SS: suspended solids; DS: dissolved solids. * Well water at a depth of 20 m; ^¥^ not detected.

## Data Availability

Not applicable.
